# Data on optimization of microprojectile bombardment parameters in development of salinity tolerant transgenic lines

**DOI:** 10.1016/j.dib.2020.105305

**Published:** 2020-02-20

**Authors:** Susmita Sarangi, Chiranjib Mandal, Sourav Dutta, Pranit Mukherjee, Raju Mondal, S.P. Jeevan Kumar, P. Ray Choudhury, Vijay Pratap Singh, Durgesh Kumar Tripathi, Asit B. Mandal

**Affiliations:** a ICAR-Central Agricultural Research Institute, Port Blair 744101, India; b ICAR-Central Research Institute for Jute and Allied Fibres, Barrackpore 700120, WB, India; c Dept. of Botany, Benaras Hindu University, Varanasi 221005, UP, India; d Indian Institute of Seed Science, Mau 275101, UP, India; e Seed Unit, Division of Crop Science, ICAR Head Quarters, New Delhi 110001, India; f C.M.P. Degree College, Constituent PG College of University of Allahabad, Allahabad 211002, UP, India; g Amity Institute of Organic Agriculture, Amity University, Noida 201313, UP, India

**Keywords:** Abiotic stress, *AmSOD* gene, Basmati rice, Microprojectile bombardment

## Abstract

This data deals with the optimization of microprojectile bombardment particles for efficient genetic transformation in an *indica* rice involving *AmSOD* gene for development of salinity tolerant transgenic lines [1]. In this study, various parameters such as effect of genotypes, helium pressure, osmoticum, explants, flight distance, particle size, particle volume, vacuum, carrier DNA and stopping screen properties have been evaluated to determine their role in transformation of *indica* rice involving *AmSOD* gene for development of salinity tolerant Pusa Basmati 1 rice variety. To perform the transformation process, plasmid vector pCAMBIA 1305.2 was used, which harbours GUS Plus™ gene, intron from the castor bean catalase gene, pBR322 ori, kanamycin resistant gene and *Xho* I site. The transformants have been confirmed using slot blot, polymerase chain reaction and Southern hybridization techniques.

**Table:** Specification Table

Subject	Section:1 Agricultural and Biological Sciences Sub section: 06, Agriculture
Specific subject area	Agricultural Biotechnology intervention made through optimization of microprojectile bombardment particles for efficient transformation in indica rice variety for enhanced tolerance to salinity.
Type of data	Table Figure
How data were acquired	Data were required using the Microprojectile instrument PDS- 1000/He system [Bio-Rad, USA (Sanford, 1993)] Based on *in vitro* culture response only one suitable variety Pusa Basmati 1 among three was selected and subjected to transgenic development involving *AmSOD* gene. Data were recorded to detect the optimized condition for maximum transient gene expression involving pCAMBIA1305.2 in respect of diverse microprojectile bombardment parameters (physical, chemical and biological). Transient gene expression data through GUS histochemical assay was used and the data were analyzed through statistical software (Microsoft Excel) [2,3]*.*
Data format	Analyzed
Parameters for data collection	In assessing the *in vitro* culture response to select the most suitable variety with prolific plantlet regeneration, data like callus induction (%), callus proliferation (w/w%), callus health (1–9 scale), shootlet regeneration (%), shootlet health (1–3 scale), root induction (%), hardening (%) and total plantlets grown to maturity were recorded. Similarly, in assessing the role of diverse physical, chemical and biological factors in influencing the transient GUS expression was assessed through GUS histochemical assay [4] involving bombarded calli with binary vector pCAMBIA1305.2 to detect the appropriate condition for microprojectile bombardment involving economically important gene *AmSOD* [5] in Pusa Basmati 1, especially at the interface of hypervariable climate.
Description of data collection	Calli were induced by culturing axenic seeds on MS medium [6] supplemented with suitable hormones, which were excised and proliferated on callus proliferation medium containing half dose of 2mg/l 2,4-D as growth regulator was used in the callus induction medium to induce fast growing embryogenic calli. Here in detecting the *in vitro* culture response to select the most suitable variety with prolific plantlet regeneration, callus induction (%), callus proliferation (w/w%), callus health (1–9 scale), shootlet regeneration (%), shootlet health (1–3 scale), root induction (%), hardening (%) and total plantlets grown to maturity were recorded. Similarly, in assessing the effect of diverse physical, chemical and biological factors in influencing the transient GUS expression was assessed through GUS histochemical assay [4] involving bombarded calli with a modern binary vector pCAMBIA1305.2 to detect the appropriate condition for successful microprojectile bombardment with maximum transformants with an important gene *AmSOD* tolerant to salinity in Pusa Basmati 1 [7].
Data source location	Institution: ICAR-Central Agricultural Research Institute, City: Port Blair 744105 Country: India Latitude and longitude: 11.6234° N, 92.7265° E
Data accessibility	Data can be accessible with related research article.
Related research article	S. Sarangi, C. Mandal, S. Dutta, P. Mukherjee, R. Mandal, S. P. Jeevan Kumar, P. R. Choudhury, V.P. Singh, D.K. Tripathi, A.B. Mandal, Microprojectile based particle bombardment in development of transgenic indica rice involving *AmSOD* gene to impart tolerance to salinity, *Plant Gene*, 19 (2019) 100183, https://doi.org/10.1016/j.plgene.2019.100183.

**Table:** 

**Value of the Data** • The datasets refers to optimization of microprojectile based particle bombardment in development of tolerant transgenic lines to salinity involving an *AmSOD* gene from heterologous mangrove plant source into the most important staple food crop rice. • This study has determined the role of transformation parameters and corroborated with *in vitro* culture response, transient gene expression, molecular analyses involving slot, Southern and PCR as well as physiological parameters, hydroponics tests, respectively. • Optimized parameters can be used for development of transgenic salt tolerant rice lines with introgression of salt tolerant genes such as *p5cs, salT, DREB, HVA1, osmotin* etc.

## 1 Data

Data pertinent to influential parameters such as effect of genotypes, helium pressure, osmoticum, explants, flight distance, particle size, particle volume, vacuum, carrier DNA and stopping screen properties have been evaluated [1] to determine their role in transformation of *indica* rice involving *AmSOD* gene for development of salinity tolerant Pusa Basmati 1 rice variety (Table 1).

**Table 1:** Synopsis of optimization experiments involving diverse factors (based on differential GUS expression) of microprojectile bombarded calli in Pusa Basmati1.

Genotype	Parameter	Range of callus size (mm^2^)	Average callus size (mm^2^)	Range of GUS spots/callus	Av No of GUS spots/callus	Range (No. GUS spots/mm^2^)	Average GUS spots/mm2	GUS staining pattern in bombarded calli^a^	Remarks
**Physical Factor**									
Pusa Basma-ti 1	**1.Helium Pressure (psi in inch)**								
	640	3.0–5.0	4.05	1–29	9.20	0.25–9.66	2.55	Faint:1.3 (26.03) Mod^b^:4.1 (36.82) Intense: 3.8 (37.09)	
	900	3.0–5.0	3.80	1–34	12.10	0.33–7.0	5.61	Faint:5.6 (45.18) Mod:2.9 (20.06) Intense:3.6 (37.74)	
	1100	3.0–5.0	3.45	0–52	15.70	0–13	4.63	Faint:2.1 (10.37) Mod:7.4 (49.35) Intense:6.2 (30.22)	**Maximum GUS expression**
	1350	2.5–4.0	3.55	0–44	11.31	0.25–8.0	2.35	Faint:3.7 (25.30) Mod:4.1 (38.08) Intense:5.8 (26.54)	
	1550	2.5–4.5	3.50	1–24	7.91	0–7.2	2.86	Faint:2.7 (33.90) Mod: 3 (42.90) Intense:2.8 (28.13)	
	**2.Particle Size (μ)**								
	0.6	2.5–5.0	3.72	2–4.5	14.81	0.57–11.25	3.76	Intense: 1.6 (10.18) Mod:7.1 (44.12) Faint:5.3 (44.60)	
	1	3.0–5.0	3.65	5–35	15.52	1.01–1.61	4.66	Intense:4.0 (27.38) Mod:14.8 (42.40) Faint: 4.2 (24.56)	**Maximum GUS expression**
	1.6	2.5–4.0	3.45	0–29	10.31	0.25–10.67	3.21	Intense:3.5 (19.09) Mod:4 (33.60) Faint:7.2 (27.20)	
	**3.Particle volume (μl)**								
	3	2.5–4.5	3.35	0–59	17.71	0–19.52	5.45	Intense:5.4 (27.30) Mod: 7.9 (49.11) Faint: 4.1 (23.59)	
	6	2.0–4.0	2.80	0–58	13.42	0–23.21	5.77	Intense:5.2 (26.40) Mod:5.0 (35.30) Faint:10.01 (65.9)	
	10	2.0–4.0	3.25	5.5–89	32.41	3.02–25.4	10.2	Intense:8.1 (47.63) Mod:7 (41.17) Faint: 2 (11.76)	**Maximum GUS expression**
	15	25.4.0	3.45	6–38	32.32	1.71–20.25	9.18	Intense: 3 (16.66) Mod:5 (28.78) Faint: 12 (66.61)	
	**4.Flight distance (cm)**								
	6	3.0–6.0	4.50	3–61	22	0.5–20.33	6	Intense:4.8 (28.63) Mod:12.0 (44.850) Faint:5.2 (26.38)	
	9	3.0–6.0	4.25	1–57	28.60	0.33–12.6	6.34	Intense: 8.1 (25.65) Mod:14.4 (56.40) Faint:3.7 (17.84)	
	12	3.0–6.0	4.05	0–39	14.01	0–8.6	3.54	Intense:2.6 (16.79) Mod:5.8 (36.56) Faint:5.6 (36.57)	
	**5.Internal vacuum pressure of chamber (Hg**″)								
	15	2–3.5	2.80	0–9	1.91	0–3	0.74	Intense: 4 (20.0) Mod:5.1 (25.00) Faint:11.02 (55.00)	
	20	2.5–4.0	3.35	0–21	7.50	0–6	1.81	Intense: 3 (15.00) Mod:5 (25.00) Faint 12.22 (60.06)	
	25	2.0–4.0	3.15	1–4	21.91	0–25	6.99	Intense: 11 (55.05) Mod:6 (30.01) Faint:4 (20.02)	
	28	2.0–3.5	2.91	0–72	31.72	0–24	10.87	Intense: 11 (52.38) Mod:6 (28.57) Faint:4.02 (19.04)	**Maximum GUS expression**
	30	2.0–4.0	3.05	0–63	30.50	0–21	9.86	Intense:6.01 (33.03) Mod: 7.02 (38.88) Faint:5.01 (27.77)	
	**6. Stopping screen position (cm)**								
	6 (Upper most)	2.0–3.5	2.63	0–74	34.50	0–22	12.2	Intense: 6.01 (33.03) Mod: 7.02 (38.88) Faint:5.01 (27.77)	
	9 (Middle)	2.0–3.0	2.44	6–57	30.01	2.4–22.8	12.5	Intense: 9 (47.36) Mod:5 (20.31) Faint:5.02 (20.31)	
	12 (lower Most)	2.0–3.0	2.75	0–79	42	0–31.62	23.84	Intense: 12 (60.00) Mod:6 (30.00) Faint:2 (10.00)	**Maximum GUS expression**
**Chemical Factor**									
	**A.0.4M Manitol**								
	4 h	2.0–4.5	2.95	0–53	19.81	0–26.51	7.93	Deep: 7.5 (28.86) Med: 6.6 (26.20) Faint: 12.7 (62.80)	
	8 h	2.02–4.5	2.90	2.47–22.11	20.10	0.66–23.5	8.86	Deep: 2.4 (12.66) Med: 5.0 (25.15) Faint: 12.7 (62.80)	**Maximum GUS expression**
	16 h	4–62	19.2	2.02–4.50	2.85	2.0–2.61	6.96	Deep:8.2 (28.68) Med: 4.0 (8.20) Faint: 7 (46.70)	
	**B.0.2M Manitol** + **0.2M Sorbitol**								
	4 h	1–31	14.91	3.0–4.5	3.56	0.22–9.66	4.36	Deep: 3.5 (38.00) Med: 5.7 (41.20) Faint4.2 (26.01)	
	8 h	2.02–4.0	3.21	0–37	14.66	0–18.51	5.41	Deep: 6.1 (31.60) Med: 3.8 (23.20) Faint: 4.8 (26.01)	**Maximum GUS expression**
	16 h	2.5–4.0	3.45	0–32	11.55	0–9.14	3.38	Deep: 2.7 (23.70) Med: 3.8 (23.60) Faint: 4.9 (39.47)	
**Biological Factor**									
	**1.Genotype**								
	Pusa Basmati 1	2.05–4.0	3.40	0–41	13.71	0–13.66	4.08	Intense:4.6 (28.72) Mod:3.6 (17.86) Faint:5.5 (38.03)	**Maximum GUS expression**
	Taraori Basmati	2.5–4.0	3.50	0–60	22.00	0–17.00	6.21	Intense: 5.2 (16.95) Mod:8.3 (38.95) Faint:8.3 (38.95)	
	IR70485-15-3-2 (NPT/Super rice)	3.0–4.5	4.05	0–64	15.50	0–14.20	3.54	Intense:6.2 (18.56) Mod:6.6 (49.02) Faint:2.7 (22.04)	
	**2.Explant**								
	Immature embryo	1.2–2	1.71	0–65	39.90	0–21.6	11.7	Intense:14.7 (25.93) Mod:15.4 (36.69) Faint:9.8 (17.96)	
	Primary Callus	2–3	2.61	9.0–46	25.52	3.0–23.0	10.36	Intense:9.3 (34.15) Mod:9.0 (34.03) Faint:7.2 (31.63)	
	Secondary Callus	1–2	1.71	4–76	26.61	2.5–57.00	16.8	Intense: 6.7 (18.10) Mod:9.4 (34.06) Faint:10.5 (43.82)	**Maximum GUS expression**
	**3.Biological Adjuvant (**Carrier DNA**)**								
	Control	2.0–3.5	2.65	4–43	22.9	1.33–17.01	9.77	Intense:9.01 (45.00) Mod:6.0 (38.00) Faint:5.01 (25.00)	**Maximum GUS expression**
	Salmon Sperm DNA	2.0–3.5	2.95	0–72	20.8	0–23.51	7.69	Intense: 7 (35.00) Mod:10 (50.00) Faint:3 (15.00)	
	Calf Thymus DNA	2.0–3.5	2.55	0–49	17.9	0–24.50	7.50	Intense: 8 (40.00) Mod:6 (30.00) Faint:6.5 (31.00)	
	Herring Sperm DNA	2.0–3.5	2.55	0–42	17.71	0–21.01	7.28	Intense:3 (15.00) Mod:10 (50.00) Faint:7 (35.00)	

a Differential GUS intensity in bombarded calli samples, figure within parenthesis indicates percent value.

b Mod: Moderate.

**Fig 1:**
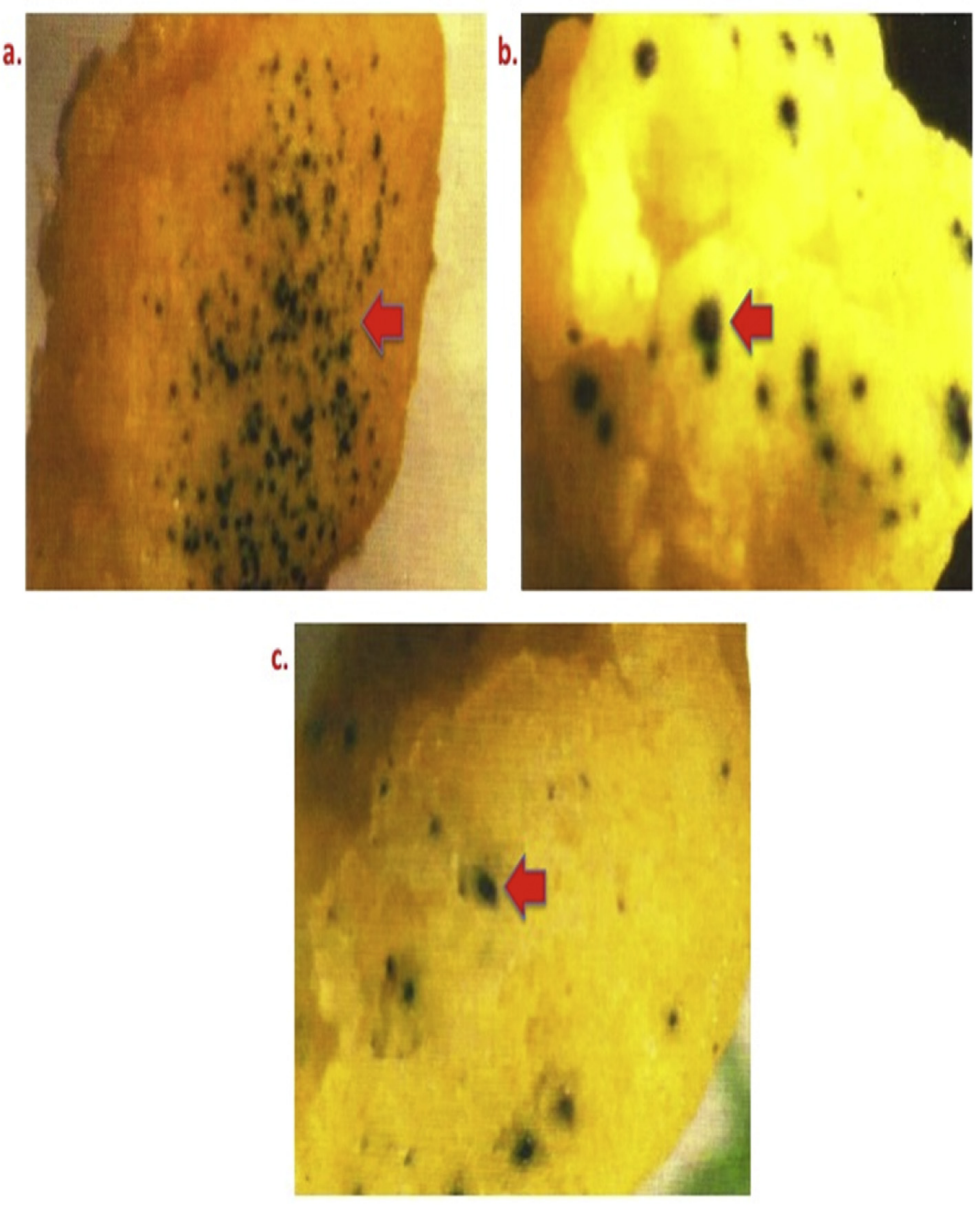
Genotypic specificity showing differential GUS expression in proliferating bombarded calli of rice using a microprojectile PDS He2000 system (Bio-Rad, USA). Legends: a) Pusa Basmati 1; b) Taraori Basmati; and c) IR70485-15-3-2 (New plant type/Super Rice).

Fig. 1 depicts the effect of genotype on transformation efficiency.

**Fig. 2:**
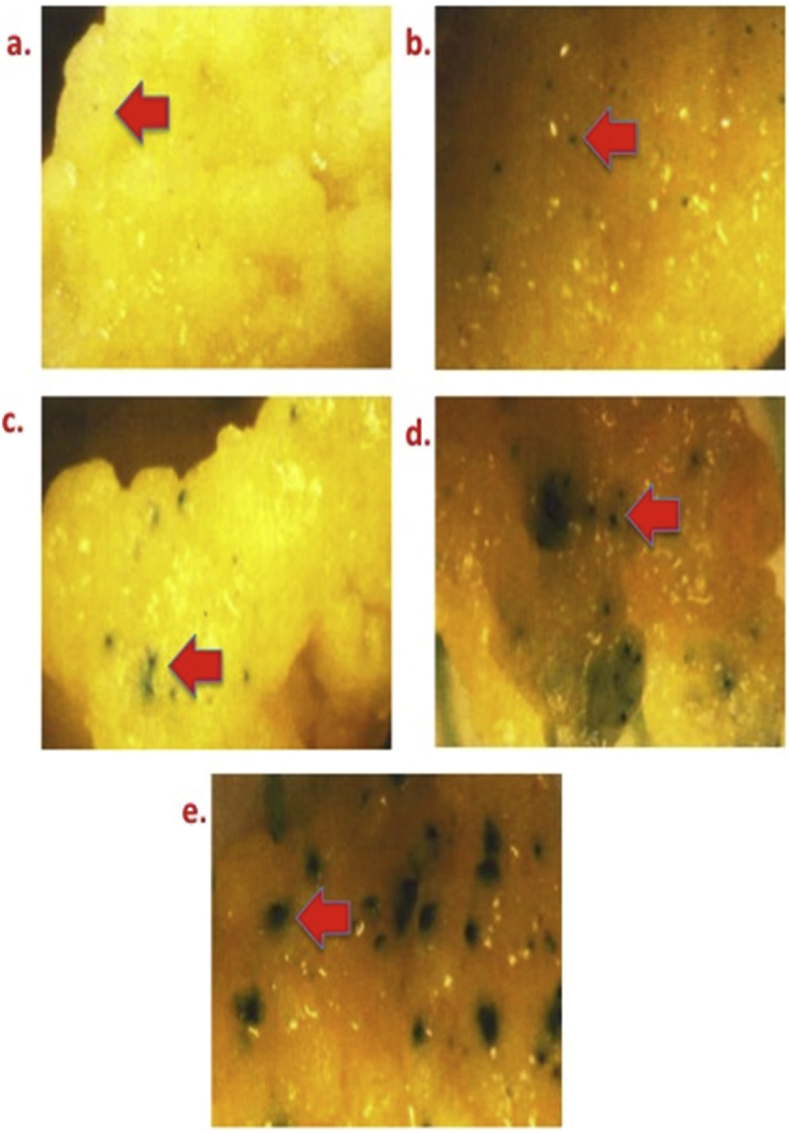
GUS expression in highly proliferating bombarded calli of Pusa Basmati 1 under different levels of He pressure in the internal chamber. Legends: a) 640 psi; b) 900 psi; c) 1100 psi; d) 1350 psi; and e)1500 psi.

Fig. 2 illustrates the role of helium pressure on transformation efficiency [a) 640 psi, b) 900 psi, c) 1100 psi, d) 1350 psi, and e) 1500 psi.

**Fig. 3:**
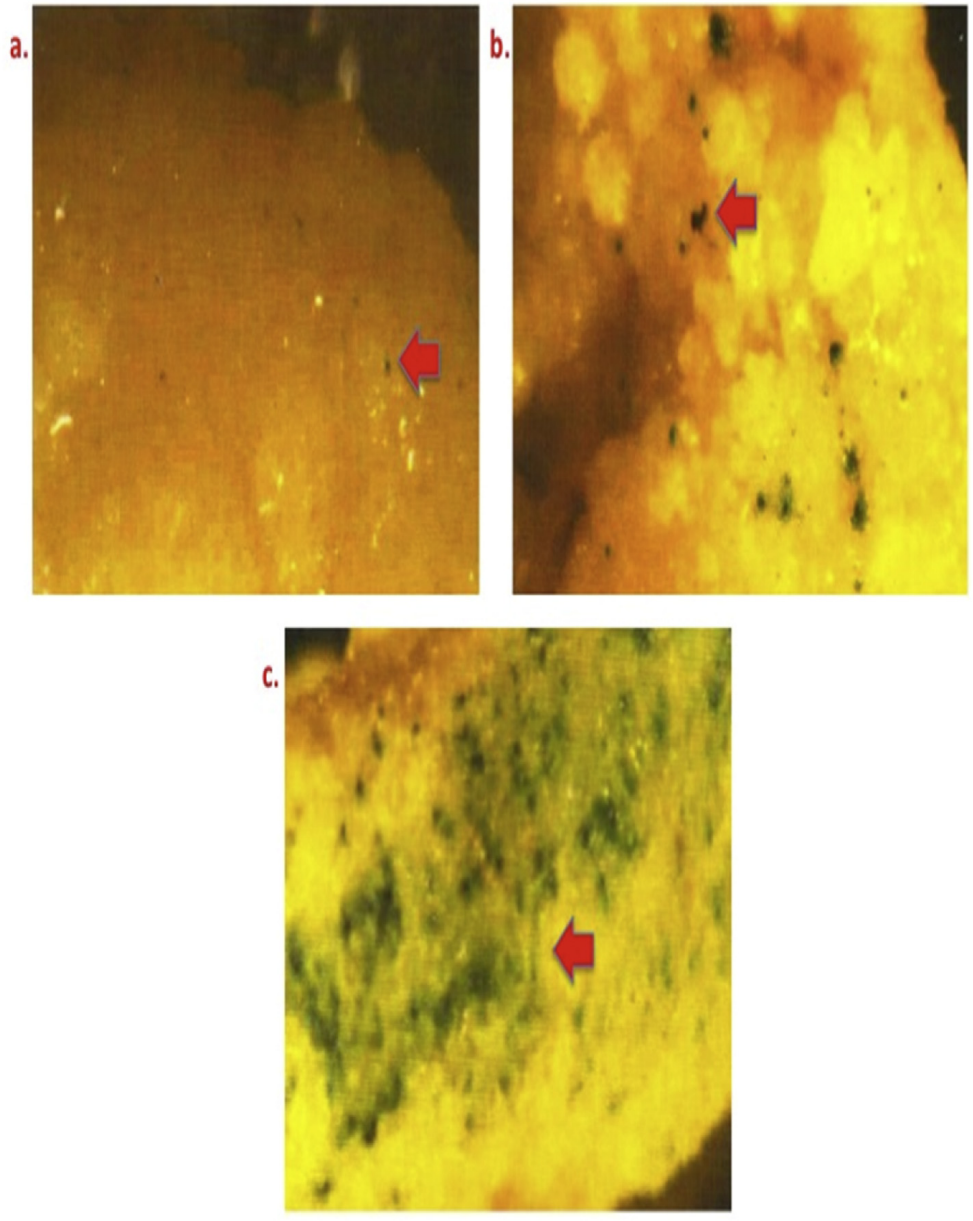
Differential GUS expression in bombarded calli of Pusa Basmati 1 at osmoticum induced by using 0.4 M mannitol singly for different time duration. Legends: a) 4 h; b) 8 h; and c) 16 h.

Effect of osmoticum (0.4 M mannitol) in inducing transformation efficiency for different period [a) 4 h, b) 8 h, and c) 16 h (Fig. 3)].

**Fig. 4:**
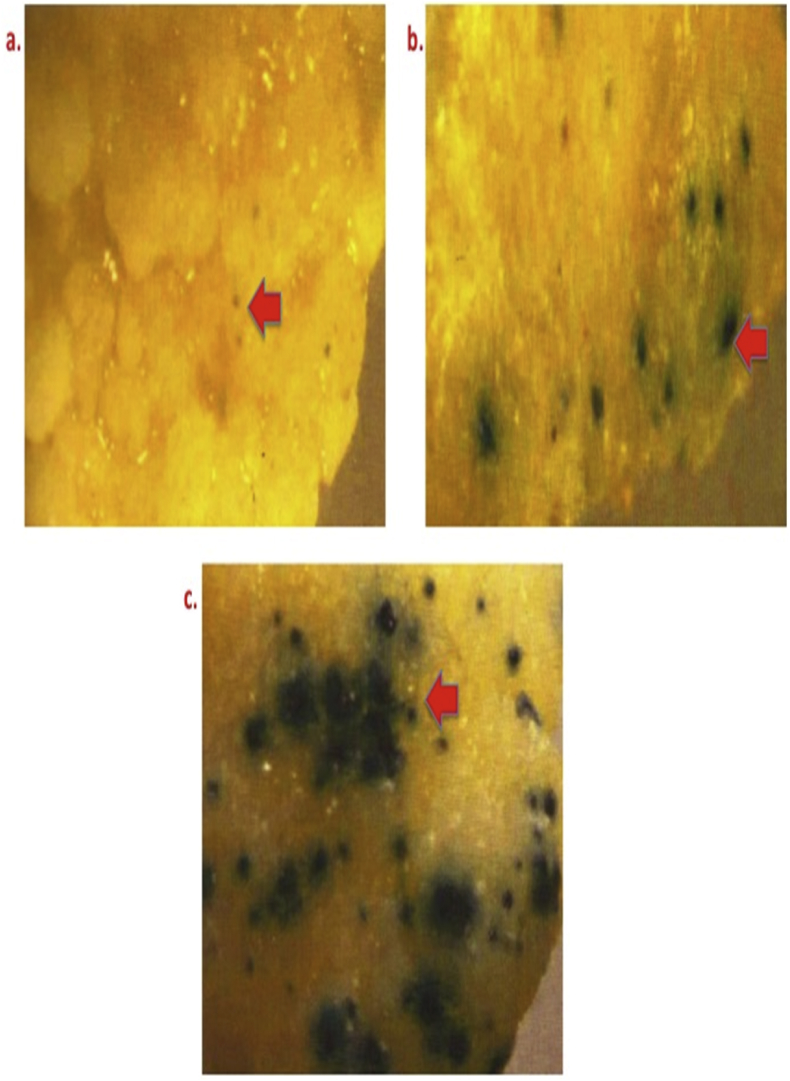
GUS expression pattern in proliferating bombarded calli of Pusa Basmati 1 at osmoticum created by using 0.2 M mannitol +0.2 M sorbitol in combination for varied time duration. Legends: a) 4 h; b) 8 h; and c) 16 h.

Fig. 4 illustrates the effect of osmoticum (0.2 M mannitol + 0.2 M sorbitol) combination for varied time duration a) 4 h; b) 8 h; and c) 16 h.

**Fig. 5:**
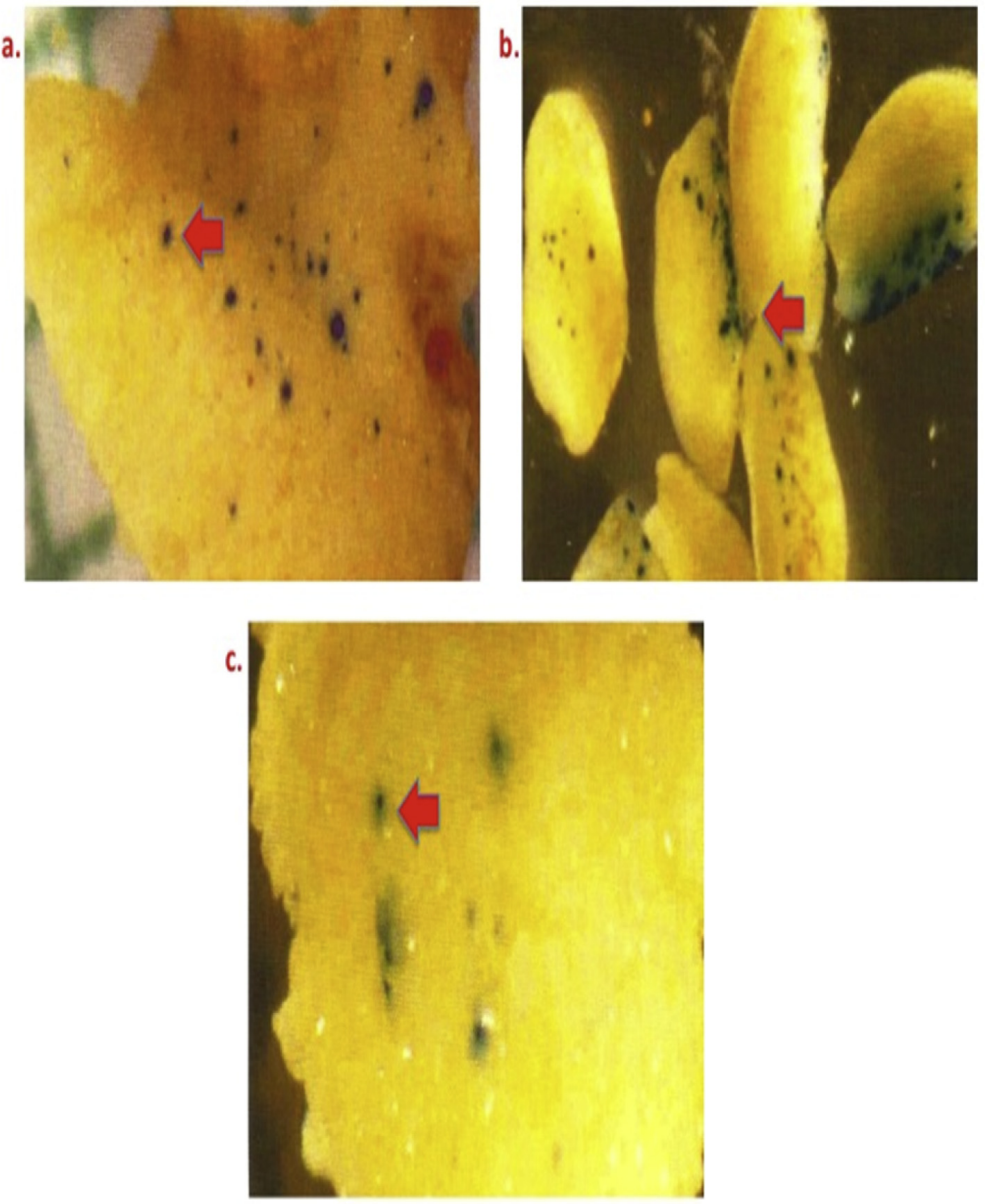
Varied GUS expression in proliferating bombarded calli derived from different explants of Pusa Basmati 1. Legends: a) Mature seed embryo derived primary callus; b)Immature embryos; and c) Mature seed embryo derived secondary callus.

Fig. 5 demonstrates the role of explants for regeneration of transformant [a) Mature seed embryo derived primary callus, b) Immature embryos, and c) Mature seed embryo derived secondary callus.

**Fig. 6:**
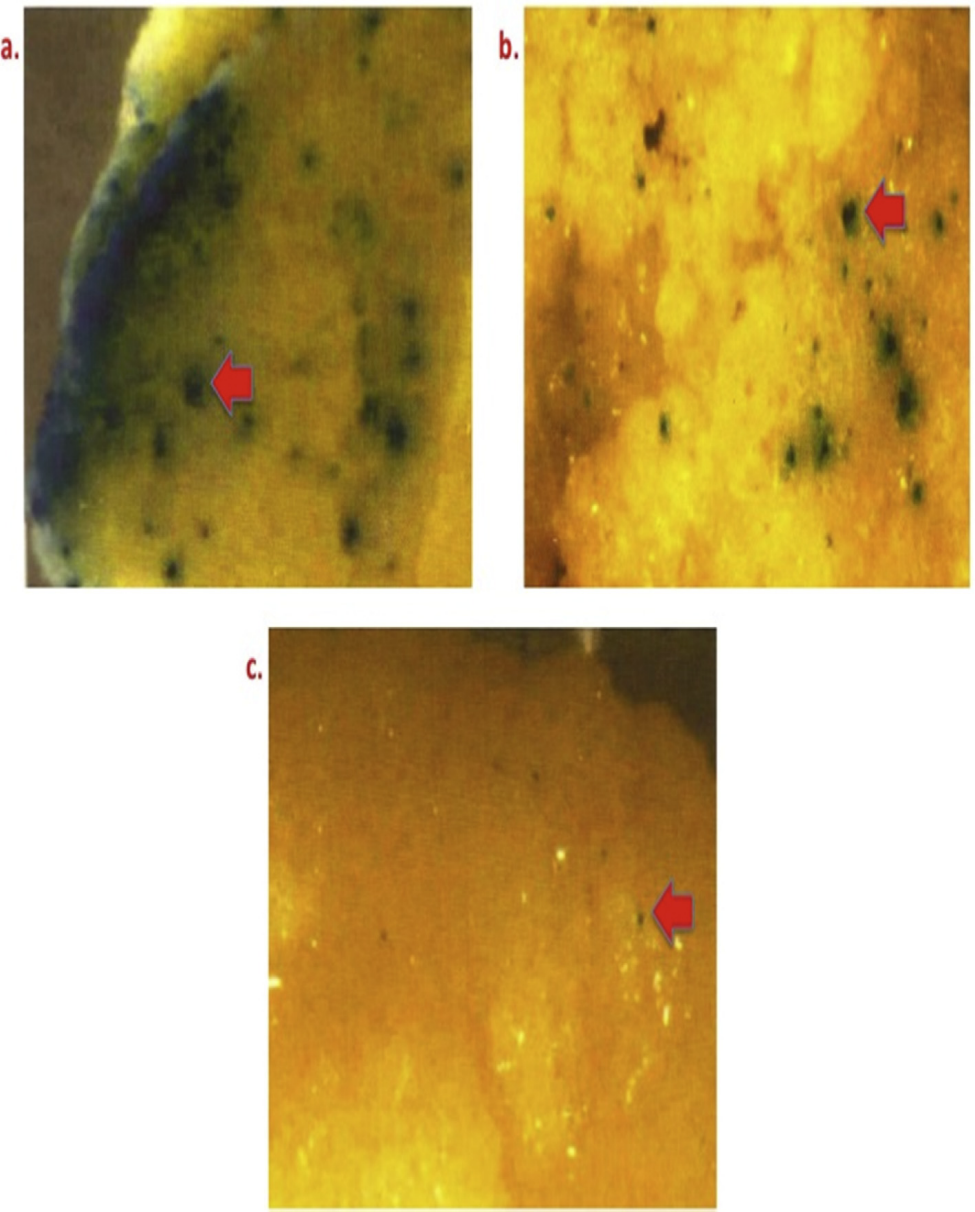
Effect of flight distance in governing GUS expression in bombarded proliferating calli of Pusa Basmati 1. Legends: a) 6 cm; b) 9 cm; and c) 12 cm.

Fig. 6 depicts the effect of flight distance in governing GUS expression of proliferating calli of Pusa Basmati 1 [a) 6 cm, b) 9 cm and c) 12 cm].

**Fig. 7:**
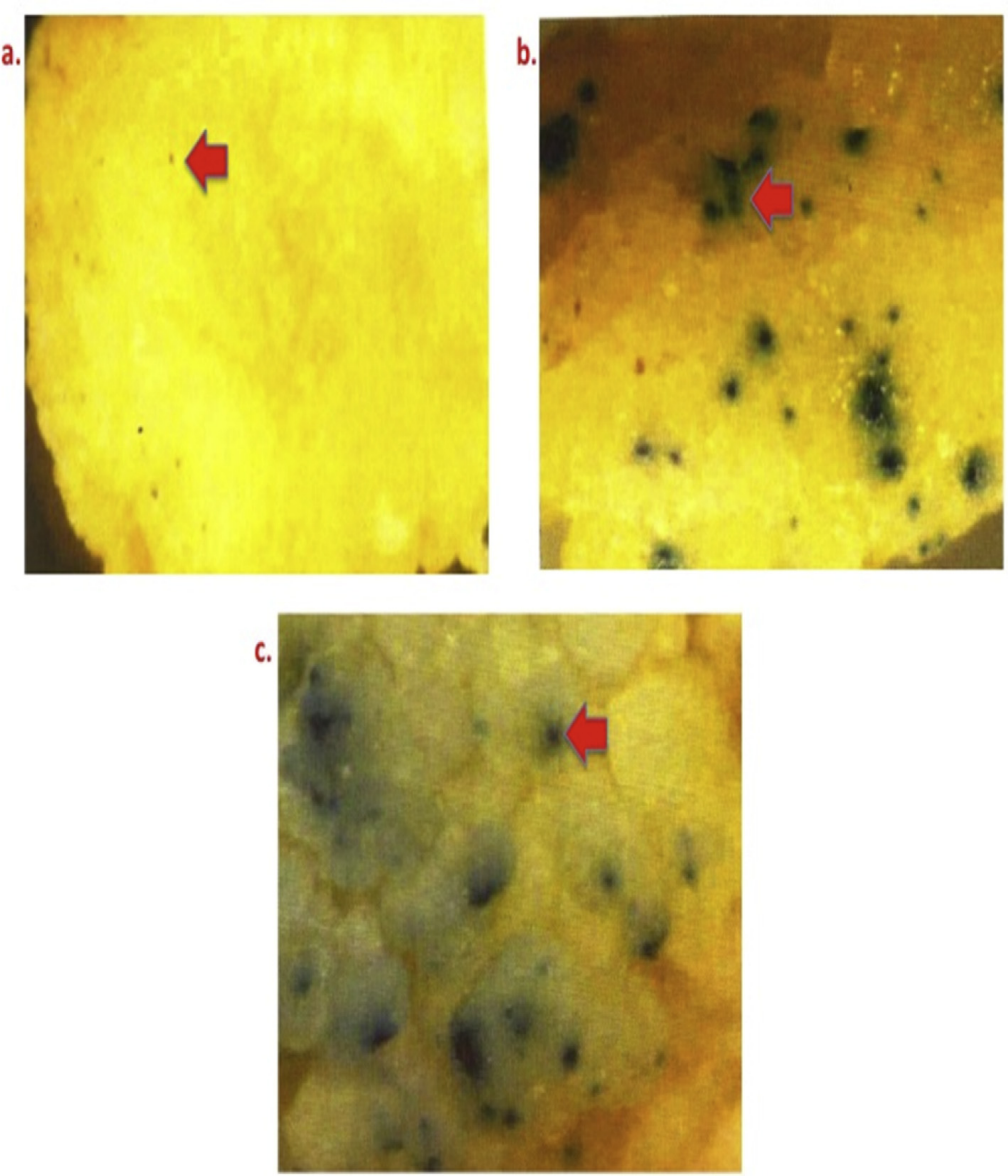
Differential GUS expression in bombarded proliferating calli of Pusa Basmati 1 using gold microcarriers of different diameter. Legends: a) 0.6 μm; b) 1.0 μm; and c) 1.6 μm.

Fig. 7 demonstrates the role of microcarrier (gold particles) in transforming the Pusa Basmati-1 [a) 0.6 μm, b) 1.0 μm and c) 1.6 μm].

**Fig. 8:**
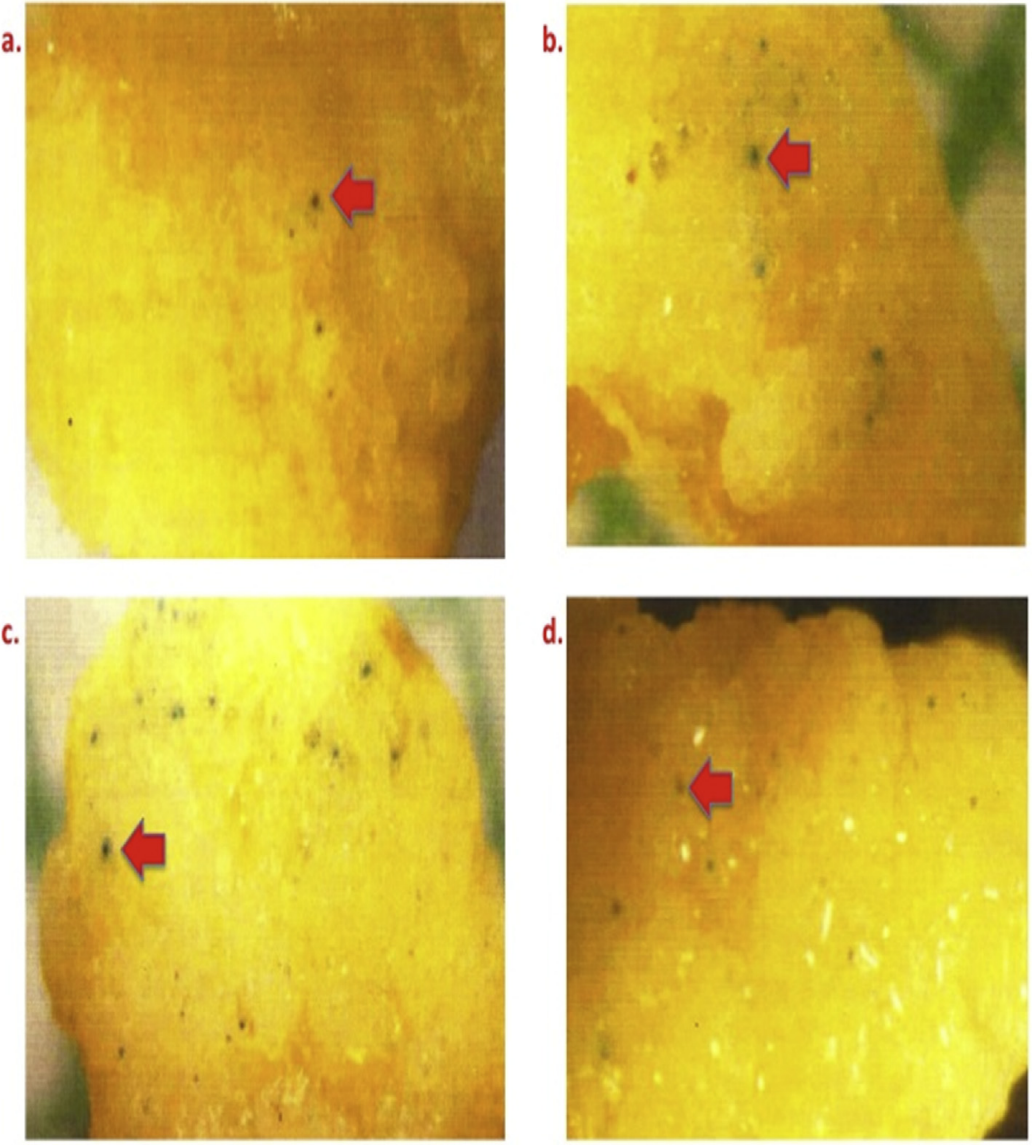
GUS expression in bombarded calli of Pusa Basmati 1 using different volumes of DNA coated microcarriers. Legends: a) 3 μl; b) 6 μl; c) 10 μl and d) 15 μl.

Fig. 8 illustrates different volumes of DNA coated microcarriers [a) 3 μl; b) 6 μl; c) 10 μl and d) 15 μl].

**Fig. 9:**
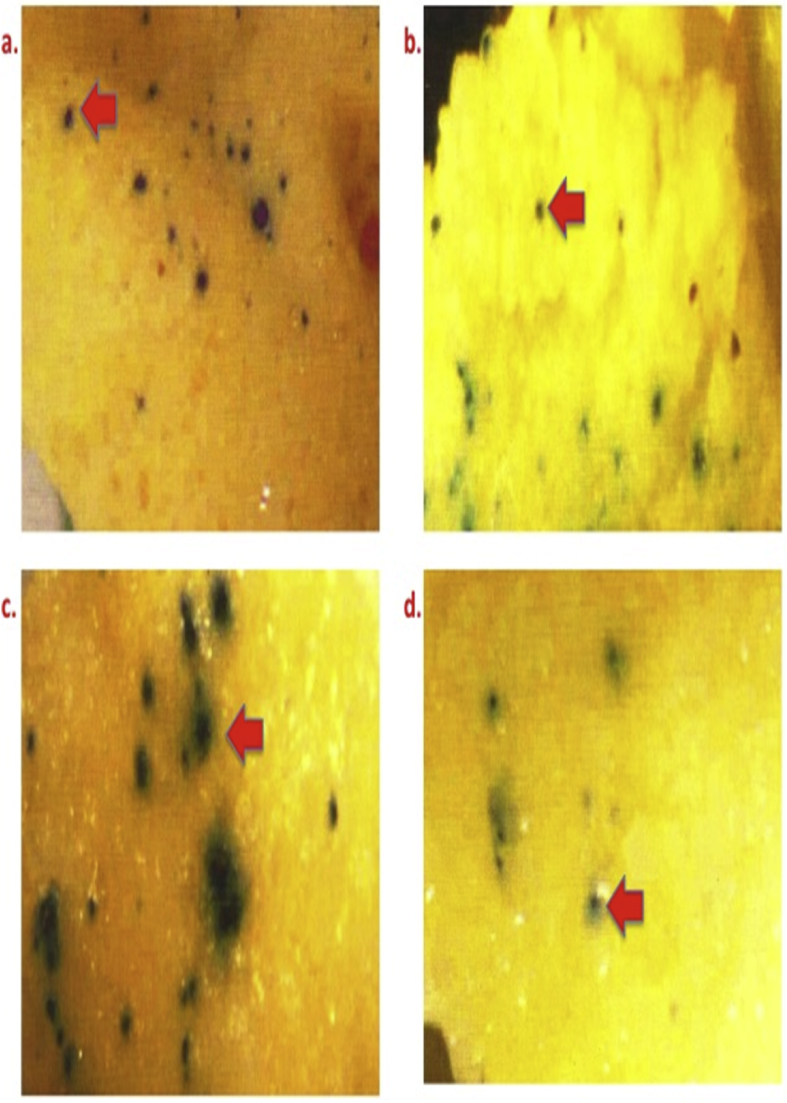
Varied GUS expression in bombarded calli of Pusa Basmati 1. Legends: a) Control; b) Salmon sperm DNA; c) Calf thymus DNA and d) Herring sperm DNA.

Fig. 9 demonstrates the GUS expression in bombarded calli of Pusa Basmati 1 [a) Control, b) Salmon sperm DNA, c) Calf thymus DNA and d) Herring sperm DNA.

## 2 Experimental design, materials, and methods

### 2.1 Effect of genotypes

To determine the effect of genotypes on transformation efficiency three genotypes have been evaluated, of which Pusa Basmati 1 was selected for further experiments (Fig. 1).

### 2.2 Effect of helium pressure

Effect of helium pressure was determined using PDS He 2000 microprojectile instrument (Bio-Rad, USA). Five pressure levels ie., 640–1550 psi have been used in bombardment of 100 randomly selected calli. After completion of 48 h the transient expression of introgressed gene has been determined by GUS expression (Fig. 2).

### 2.3 Effect of osmoticum

To reduce cell viability and sometime necrosis, osmoticum like manitol (Sigma-Aldrich Cat No. M4125) was used to increase the osmolarity. 0.4 M manitol supplemented in the bombardment medium was used for culture of primary calli (size 2.0–4.5 mm^2^ with average of 2.95 mm^2^) of all three varieties for 4, 8,16 h (Table 1 and Fig. 3). Dual osmoticum treatments with manitol and sorbitol (0.2 M) were also studied for pre-incubation as shown in (Table 1and Fig. 4).

### 2.4 Effect of explants

To identify most suitable one, different explants were bombarded and GUS expression was recorded under stereo zoom microscope (Nikon, Japan) (Table 1). Secondary calli was used as suitable explants for microprojectile bombardment for transgenic development (Fig. 5).

### 2.5 Effect of flight distance

Microcarrier velocity is intimately related to air resistance that governs transgene delivery and its expression in the recipient system. In the present experiment three flight distances viz. 6, 9, and 12 cm were used to bombard primary calli of 3.0–6.0 cm size with average of 4.5 cm (Table 1 and Fig. 6).

### 2.6 Effect of particle sizes

Inert gold microcarriers of three different diameters viz. 0.6, 1.0, and 1.6 μm (Bio-Rad, USA) were used to achieve maximum transgene delivery and transient expression of the reporter gene involving primary calli [size range: 2.5–5.0 mm^2^ (average: 3.7 mm^2^)] (Table 1 and Fig. 7).

### 2.7 Effect of particle volume

In the present study, four volumes viz. 3,6,10 and 15 μl particle volume were used to bombard primary calli (range: 2.5–4.0 mm^2^; average: 3.35 mm^2^) (Table 1 and Fig. 8).

### 2.8 Effect of vacuum

In this experiment, 5 internal vacuum pressure viz. 15″, 20″, 25″, 28″ and 30″ of Hg were used in bombarding proliferative calli of the size range: 2.02–3.5 mm (average: 2.8 mm^2^) (Table 1).

### 2.9 Effect of stopping screen position

In this study, bottom, middle and top most positions for placement of stopping screen were used in bombarding calli of size range: 2.0–3.5 mm^2^ and average: 2.6 mm^2^ (Table 1).

### 2.10 Effect of carrier DNA

To safe guard the intruded DNA from endogenous nuclease inside the recipient calli, Salmon Sperm DNA, Calf thymus DNA, Herring sperm DNA were used as coating material of the microcarriers along with plasmid DNA (pCAMBIA 1305.2 ref.website) in 1:1 ratio. Primary calli of 2.0–3.0 mm size with average of 2.5 mm^2^ diameters were bombarded (Fig. 9) (Table 1).

## References

[1] Sarangi S., Mandal C., Dutta S., Mukherjee P., Mandal R., Jeevan Kumar S.P., Choudhury P.R., Singh V.P., Tripathi D.K., Mandal A.B. (2019). Microprojectile based particle bombardment in development of transgenic indica rice involving *AmSOD* gene to impart tolerance to salinity. Plant Gene.

[2] Klein T.M., Gradziel T., Fromm M.E., Sanford J.C. (1988). Factors influencing gene delivery into *Zea mays* cells by high–velocity microprojectiles. Nat. Biotecnol..

[3] Petrillo C.P., Carneiro N.P., Purcino A.Á.C., Carvalho C.H.S., Alves J.D., Carneiro A.A. (2008). Optimization of particle bombardment parameters for the genetic transformation of Brazilian maize inbred lines. Pesqui. Agropecu. Bras..

[4] Jefferson R.A. (1987). Assaying chimeric genes in plants: the GUS gene fusion system. Plant Mol. Biol. Rep..

[5] Sarangi S., Ghosh J., Bora A., Das S., Mandal A.B. (2011). *Agrobacterium*-mediated genetic transformation of *indica* rice varieties involving Am-SOD gene. Indian J. Biotechnol..

[6] Murashige T., Skoog F. (1962). A revised medium for rapid growth and bio-assays with tobacco tissue cultures. Physiol. Plant.

[7] Jeevan Kumar S.P., Rajendra Prasad S., Banerjee R., Thammineni C. (2015). Seed birth to death: dual functions of reactive oxygen species in seed physiology. Ann. Bot..

